# Protein‐Independent Liquid–Liquid Phase Separation of Adenosine Triphosphate Under Crowded Conditions

**DOI:** 10.1002/chem.71374

**Published:** 2026-07-03

**Authors:** Robert Dec, Wojciech Dzwolak, Roland Winter

**Affiliations:** ^1^ Physical Chemistry I – Biophysical Chemistry, Department of Chemistry and Chemical Biology TU Dortmund University Dortmund Germany; ^2^ Faculty of Chemistry, Biological and Chemical Research Centre University of Warsaw Warsaw Poland

**Keywords:** adenosine triphosphate, biophysics, chemistry, circular dichroism, cotton effect, ionic strength, intracellular, macromolecular crowding, phase transition, polyethylene glycol

## Abstract

Adenosine triphosphate (ATP), a common constituent of protein‐rich biomolecular condensates, itself undergoes liquid–liquid phase separation (LLPS) even in the absence of polypeptides. Using polyethylene glycol as macromolecular crowding agent, we observed robust ATP droplet formation at pH 2–11. Moderate NaCl concentrations and lower temperatures promote LLPS by lowering critical ATP and crowder concentrations. While very high salt reverses this trend, the droplets nevertheless survive in hypersaline environments. We argue that both ion‐mediated bridging and anomalous underscreening likely contribute to the response of ATP droplets to increasing ionic strength. Most importantly, physiological millimolar ATP concentrations are sufficient for phase separation in the presence of millimolar Mg^2^^+^ and crowders, mimicking intracellular conditions. pH tunes intermolecular interactions, evidenced by inversion of the adenosine circular dichroism (CD) signal. These results reveal intrinsic, protein‐independent LLPS of ATP with potential roles in cellular compartmentalization and pathological phase transitions.

## Graphical abstract

ATP itself is able to undergo liquid–liquid phase separation in the presence of the macromolecular crowding agent PEG and physiological ATP concentrations are sufficient for phase separation in the presence of mM Mg^2^^+^ and crowders, mimicking intracellular conditions. These results reveal intrinsic, protein‐independent liquid–liquid phase separation of ATP with potential roles in cellular compartmentalization.

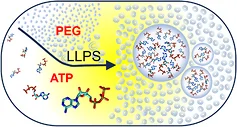

## 1 Introduction

Liquid–liquid phase separation (LLPS) is a ubiquitous physicochemical process that drives the assembly of membraneless organelles such as nucleoli, P bodies, and stress granules [1, 2, 3, 4, 5, 6, 7]. It has also been proposed that LLPS enabled the emergence of prebiotic protocells by furnishing a primitive yet effective route to compartmentalization [8, 9, 10, 11]. Conversely, dysregulated protein phase separation is increasingly associated with the early stages of pathological aggregation in neurodegenerative diseases and beyond, such as by α‐synuclein, FUS, tau and PrP [12, 13, 14, 15, 16, 17, 18, 19, 20].

Adenosine triphosphate (ATP), best known as the universal energy currency of the cell, also exerts profound effects on biomolecular condensation. The molecule exhibits an amphiphilic nature due to the presence of both charged, hydrophilic groups, the triphosphate, and a hydrophobic adenosine ring system. At millimolar physiological concentrations, ATP can either promote or suppress protein phase separation, depending on the system. While the mechanisms by which ATP affects proteins in solution are nuanced and still under debate, the nucleotide often acts as a potent hydrotrope that solubilizes hydrophobic polypeptides, inhibits amyloid formation, and dissolves pre‐existing aggregates, but may also promote aggregation of neutral macromolecules, that is, show salting‐out behavior [21, 22, 23, 24]. In other contexts, ATP actively drives LLPS by bridging multivalent cationic proteins and peptides through electrostatic and cation–π interactions, as observed with FUS [25, 26], arginine‐ [27] or lysine‐rich [28, 29, 30, 31] peptides, IgG1 antibodies [32], and the N‐protein of SARS‐CoV‐2 [33]. Depending on its concentration, ATP can promote either type of transition [25, 34]. In all cases reported to date, however, ATP acts as a minor component or modulator within protein‐ or peptide‐rich condensates. Like many amphiphilic molecules in aqueous solution, ATP tends to self‐associate at the molecular and microscopy levels or forms nanoclusters as revealed by NMR‐monitoring [24, 35, 36, 37, 38, 39, 40]. However, the observation reported here of phase‐separating liquid droplets formed by ATP in the absence of proteinaceous partners under molecular crowding conditions (known to favor droplet formation [41, 42, 43, 44, 45, 46, 47], a topic excellently reviewed by André and Spruijt [49]) emerged unexpectedly. This intrinsic, protein‐independent phase behavior of ATP reveals a hitherto unrecognized dimension of this quintessential metabolite and raises new questions about its potential roles in the formation of cellular condensates, their regulation, and pathology‐associated phase transitions.

## 2 Results and Discussion

### 2.1 Crowder‐Induced Phase Separation and Droplet Formation of ATP

The discovery reported here—that ATP alone, without any protein, peptide, or nucleic acid, undergoes robust liquid–liquid phase separation under conditions of macromolecular crowding—was serendipitous. Routine microscopy inspection of mixed aqueous ATP‐PEG samples used as control in a different project (whose results have been published recently [50]) revealed the presence of highly dynamic droplets (Figure 1A and Movies S1 and S2). The droplets formed rapidly in a very broad pH range (from 2 to 11) as long as the concentrations of both ATP and PEG were above certain critical levels. Optical density (turbidity) measurements at 500 nm were selected as a high‐throughput means of quantifying the abundance of droplets. The corresponding plots are shown in Figure 1B,C. In concentrated (33.3% wt./vol.) PEG‐3350 solution at 21°C, LLPS occurred at ATP concentrations above 55 mg/mL at pH 7 or 35 mg/mL at pH 4, clearly indicating that the protonation‐induced decrease in repulsion between unscreened ATP ions favors droplet formation. The phase separation showed an upper critical solution temperature (UCST) phase behavior: raising the temperature from 21°C led to mixing, while cooling the samples prepared at subcritical concentrations triggered demixing below ∼10°C (Figures 1D,E and S1).

**FIGURE 1:**
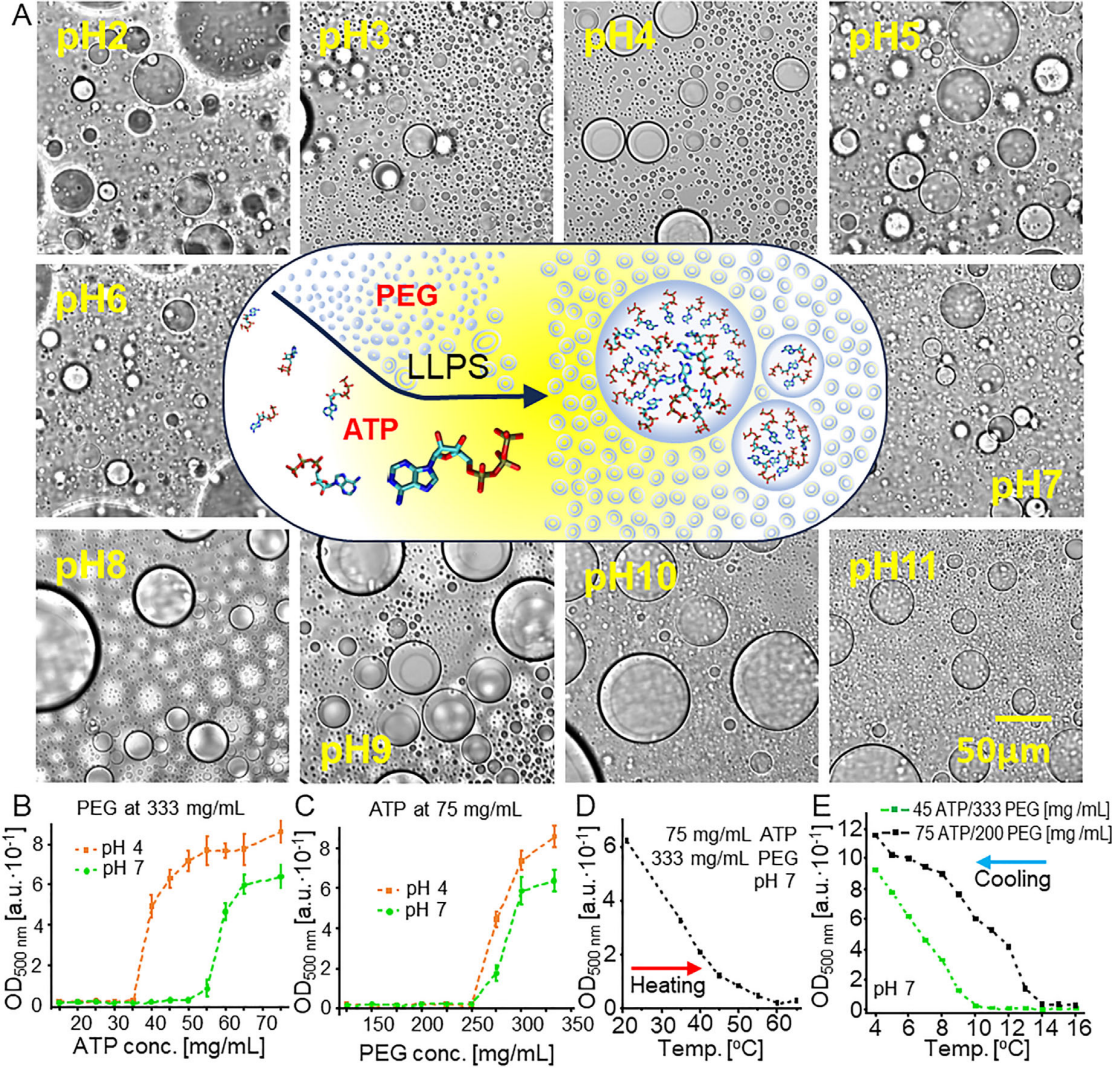
Under molecular crowding conditions, ATP forms liquid droplets on its own in a broad pH range. Brightfield microscopy images of droplets formed in aqueous solutions containing 75 mg/mL ATP and 333 mg/mL PEG‐3350 (average MW of 3350 Da) with pH adjusted to the indicated values; all images were acquired at room temperature (A). Optical density (absorbance at 500 nm) arising from the LLPS as a function of ATP concentration (at fixed 333 mg/mL PEG‐3350 concentration—B) and of PEG‐3350 concentration (at fixed 75 mg/mL ATP concentration—C) at pH 4 and 7 and at 21°C. Temperature‐induced mixing in the two‐phase system formed in the presence of 75 mg/mL ATP and 333 mg/mL PEG‐3350, pH 7, probed by absorbance measurements at 500 nm (D). Cooling‐induced LLPS in the single‐phase systems with initial subcritical concentrations of ATP (45 ATP/333 PEG [mg/mL]) and PEG‐3350 (75 ATP/200 PEG [mg/mL]), pH 7 (E).

Under the various conditions investigated here, the ATP droplets coalesced rapidly (see Movies S1 and S2) and formed a macroscopically separated coacervate phase (Figure 2A), which accounted to a lesser fraction the total volume (subject to the pH conditions and the exact mixing stoichiometry—see Table S1). The segregated system is quite stable even under ambient conditions (see Figure S2). The different ATP concentrations in the upper and bottom liquid phases were estimated spectrophotometrically at three different pH values (see inset Table in Figure 2A), the nucleotide concentration in the bottom layer is at least one order of magnitude higher than in the upper phase and continues to increase as the pH decreases (∼320 mg/mL at pH 9 compared to ∼390 mg/mL at pH 7 and to ∼437 mg/mL at pH 4).

**FIGURE 2:**
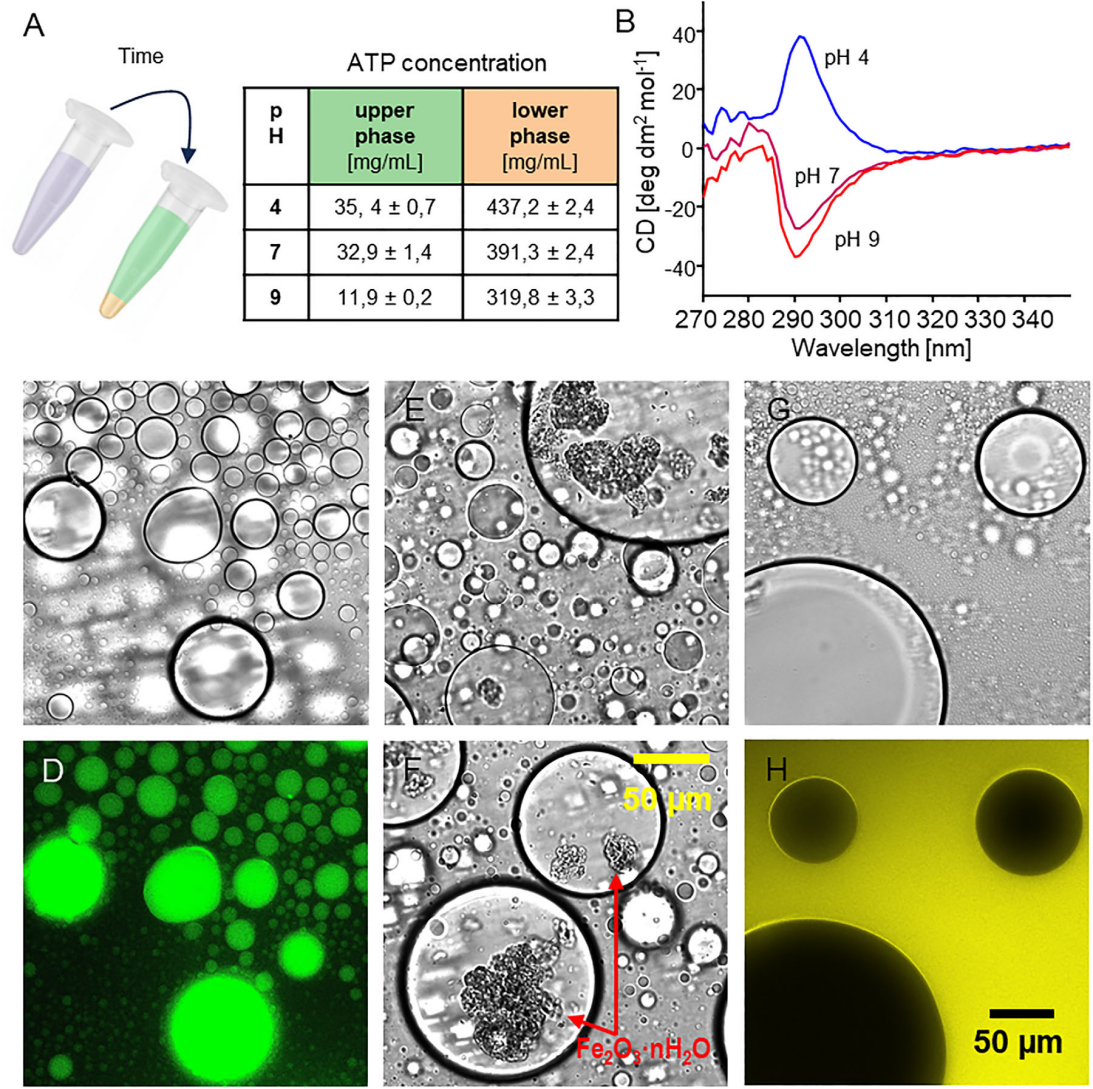
Composition and properties of the ATP droplets. Droplets formed in aqueous mixture of 75 mg/mL ATP and 333 mg/mL PEG‐3350, pH 7, readily coalesce forming two macroscopic phases of which the bottom is the ATP‐rich phase (A). Table inset: concentrations of ATP in the ATP‐rich and top phases at pH 4, 7, and 9, obtained from spectrophotometric measurements (at 260 nm) of water‐diluted aliquots. CD spectra of centrifuged ATP‐rich phases at pH 4, 7, and 9 (75 mg/mL ATP + 333 mg/mL PEG‐3350, 25°C), measured using a 0.01 mm optical pathway and subsequently normalized to the ATP concentration (B). Brightfield (C, E, G, F) and fluorescence (D, H) microscopy images of 75 mg/mL ATP, 333 mg/mL PEG‐3350, pH 7 droplets containing 0.02 mg/mL quinacrine (C, D), microparticles of sonicated Fe_2_O_3_·nH_2_O (E, F) or 0.2 mg/mL fluorescent PEG‐FITC (G, H). The fluorescence was excited at excitation 480/40 nm and at emission 527/30 nm, both for PEG‐FITC and quinacrine.

Circular dichroism (CD) spectra of the ATP‐rich bottom phases at these three different pH values were collected using a quartz cuvette with an extremely short optical pathway of 0.01 mm (Figure 2B). We noted that the sign of the Cotton effect induced in the adenine chromophore switches from negative to positive when the pH is lowered from 7 to 4, indicating distinct patterns of intermolecular association and hence transition dipole moment‐couplings between the ATP molecules, which is reminiscent of a similar behavior of solution‐phase ATP oligomers [36].

### 2.2 Partitioning of Cosolutes in ATP Droplets

We have subsequently used brightfield and fluorescence microscopy to probe the droplets’ capacity to sequester selected foreign molecules and particles (Figure 2C–H). While fluorescent quinacrine (Figure 2C,D), and to some extent inorganic Fe_2_O_3_·nH_2_O microparticles [51] readily accumulate within the ATP droplets (Figure 2E,F), fluorophore‐labelled PEG does not enter the droplets, as shown in Figure 2H. Thus, PEG is completely excluded from the ATP‐rich droplet phase (see, Figure S3, for quantitative estimation of partition coefficients of both quinacrine and PEG‐FITC).

### 2.3 Salt‐Effects on Phase Separation and Droplet Formation of ATP—Screening, Ion‐Bridging and Underscreening

As the interplay of hydrophobic, π‐stacking, and Coulombic interactions clearly affects the stability and dynamics of ATP droplets, we investigated the LLPS transition in the presence of moderately concentrated NaCl, which provides Debye screening for the repulsive forces between nucleotide polyanions. The optical density traces and microscopy images in Figure 3 show how the addition of 2 M NaCl lowers the critical concentrations of ATP and PEG (panels A and B, respectively) at which LLPS occurs. The effect of salt at this concentration is profound: when all other parameters (except for ionic strength) are kept constant, the droplet state becomes accessible to ATP at concentrations approximately 4 times lower. Likewise, the critical concentration of PEG is reduced by about 40% (Figure 3B compared to Figure 1C).

**FIGURE 3:**
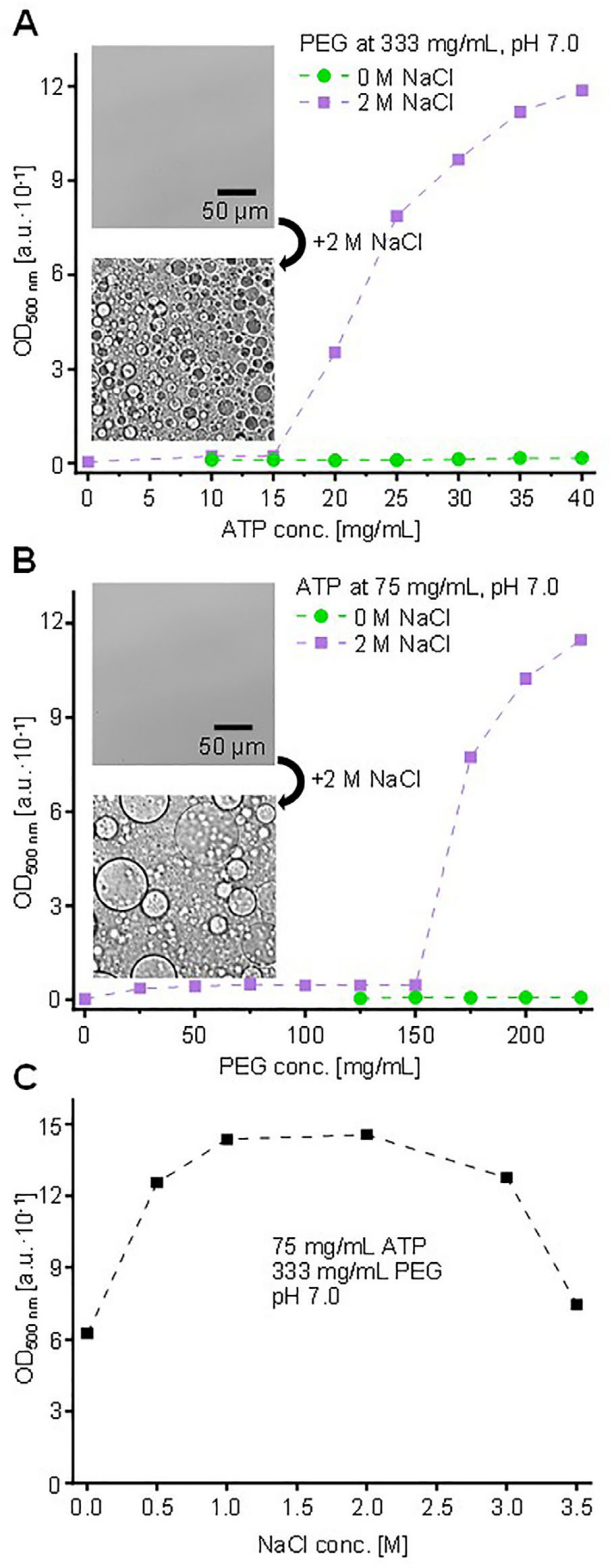
Impact of high ionic strength on LLPS in the ATP‐PEG‐3350 system. Optical density changes accompanying titration of PEG (333 mg/mL, pH 7) with ATP (A) and vice versa (75 mg/mL ATP, pH 7 – B) in the absence and presence of added 2 M NaCl (both at 21°C). Microscopy images of liquid 25 mg/mL ATP + 333 mg/mL PEG in the absence and presence of 2 M NaCl are presented in panel A, whereas the inset in panel B shows the analogous images collected for 75 mg/mL ATP + 175 mg/mL PEG (i.e., at subcritical for LLPS concentrations of ATP and PEG, respectively). Nonmonotonic dependence of the optical density of the 75 mg/mL ATP + 333 mg/mL PEG, pH 7 system on the concentration of added NaCl (C).

Interestingly, a more comprehensive investigation of the effect of NaCl concentration on LLPS in the ATP‐PEG system (probed by turbidity; Figure 3C) reveals a nonmonotonic response, where the droplet state becomes relatively less favored at salt concentrations exceeding 2 M NaCl. This complex behavior suggests a nuanced interplay between disparate electrostatic forces. While π‐stacking interactions remain the primary drivers of ATP self‐association [25], the initial enhancement of ATP phase separation at low [NaCl] may be attributed to counterion (Na^+^) bridging negatively charged ATP moieties. Recent studies on highly charged biomolecules, such as highly charged and intrinsically disordered Caprin1 protein [52], have demonstrated that counterions can facilitate effective attractions between like‐charged molecules by forming bridges that outweigh conventional Debye‐Hückel screening at low ionic strengths [52, 53]. As the salt concentration increases beyond the optimal regime for bridging, conventional screening begins to dominate, leading to the observed reentrant decrease in phase separation propensity. This effect may be further exacerbated at high ionic strengths, where conventional electrostatic models give way to the nonclassical phenomenon of anomalous underscreening. In this regime, the electrostatic decay length becomes significantly larger than predicted by classical mean‐field theory [54, 55, 56]. By causing a decrease in effective screening, this phenomenon potentially leads to the re‐establishment of long‐range repulsion between ATP molecules that was previously suppressed by counterion bridging. We note, however, that ATP droplets still persist even at the highest recorded salt concentration (3.5 M NaCl, see also Figure S4), suggesting they could survive hypersaline environments, such as those found in terrestrial or Martian subsurface salt lakes [57], potentially serving as primitive compartments in prebiotic evolution.

The initial droplet‐stabilizing bridging effect is expected to be more pronounced with divalent cations as their higher valency reduces the entropic penalty associated with counterion sequestration into the condensate [52]. Consistent with this rationale, we further examined ATP LLPS by replacing NaCl with MgCl_2_.

The data presented in Figure 4 indicate that millimolar concentrations of Mg^2+^ cations, which naturally complex with cellular ATP, make the LLPS transition even more accessible than nonphysiologically high concentrations of NaCl. Specifically, at 50 mM MgCl_2_, ambient temperature, and neutral pH, droplets begin to form at a concentration of 2.5 mg/mL ATP (∼ 5 mM), which is within the physiological range [57, 58, 59] (see the sparse complex droplets in the left section of Figure 4A). Of note, ATP concentrations can reach much higher values in specific cellular compartments, such as 0.1 M in the adrenal medulla [24]. The increased droplet formation continues even when the Mg^2+^ concentration is reduced to 30 mM (Figure 4B). Moreover, we observed that tiny dispersed droplets form near the glass‐solution interfaces even when the PEG concentration is reduced to 17.5% wt./vol. (at 30 mM MgCl_2_ and 7 mg/mL ATP, pH 7) and the temperature is reduced to 4°C (Figure 4C). One of the characteristic features of Mg^2+^‐stabilized ATP droplets is their persistence in solution in the form of a finely dispersed emulsion that coalesces into the macroscopically separating phase over much longer periods of time than the droplets formed in the absence of Mg^2+^ and at high nucleotide concentration (Figure 4D). The higher efficiency of divalent Mg^2+^ counterions in maintaining the droplet state at the low concentrations clearly resonates with the ion‐bridging argument [52].

**FIGURE 4:**
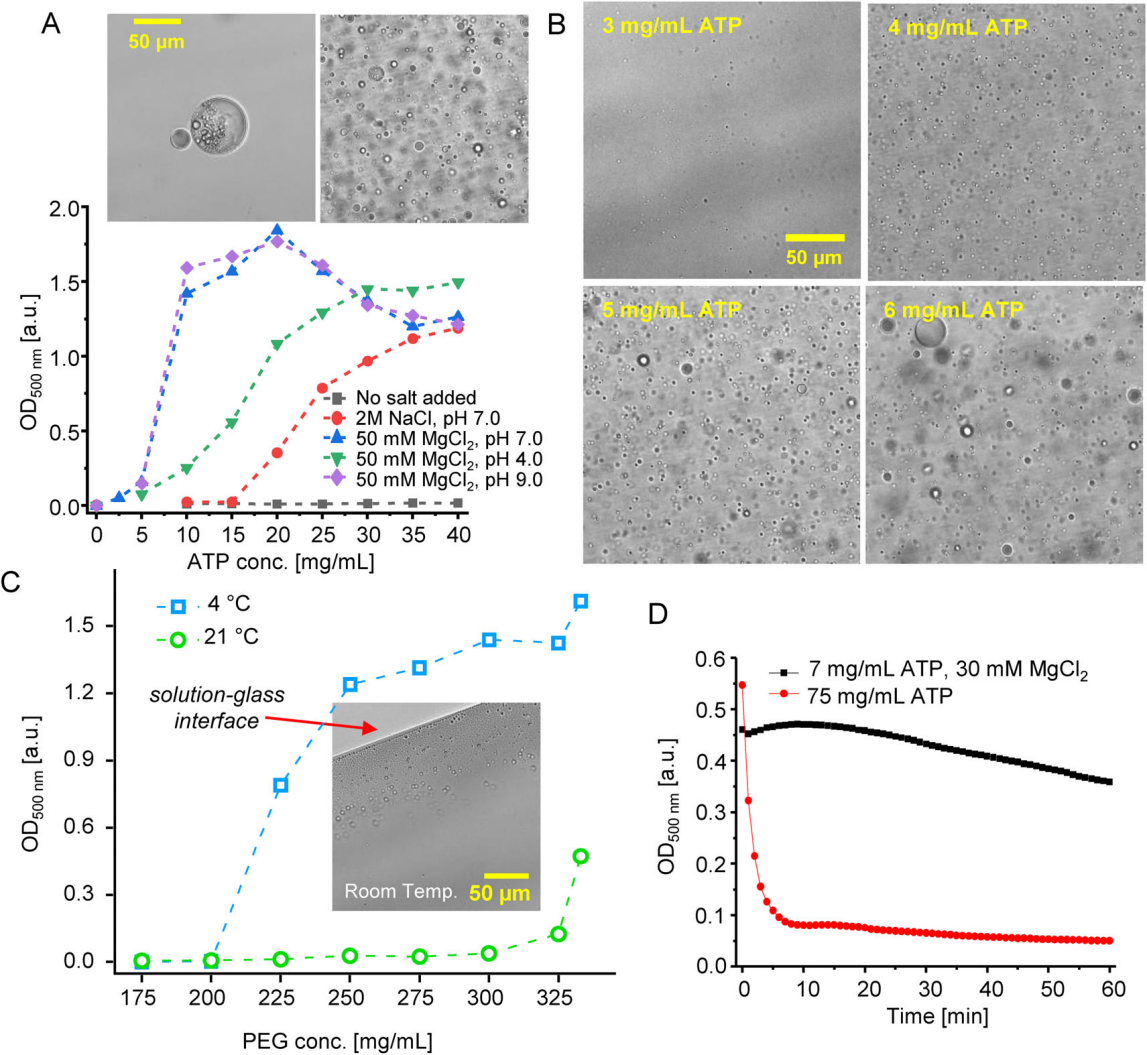
Moderate concentrations of Mg^2+^ ions shift the LLPS transition to the physiological range of ATP concentrations. Course of LLPS‐triggered changes in optical density at 21°C with increasing ATP concentration in the absence and presence of 2 M NaCl, and in the presence of 50 mM MgCl_2_ at pH 4, 7, and 9; the PEG‐3350 conc. being 333 mg/mL (A). The left and right microscopy images shown in the inset correspond to samples containing initially subcritical ATP concentrations (2.5 and 10 mg/mL, respectively) with 50 mM MgCl_2_, pH 7, and PEG‐3350 at 333 mg/mL. Brightfield images of droplets stabilized by 30 mM MgCl_2_ and 333 mg/mL PEG‐3350, pH 7, of ATP at various concentrations in the close‐to‐cellular range (B). Optical density dependency on PEG concentration in the Mg^2+^‐containing ATP samples (7 mg/mL ATP, 30 mM MgCl_2_, pH 7) at 21°C and 4°C (C). The inset image shows how tiny droplets are formed at the solution‐glass interface even at room temperature and subcritical PEG concentration of 175 mg/mL. Tiny Mg^2+^‐stabilized droplets coalesce more slowly than droplets formed at high ATP concentrations, as reflected by different rates of optical density changes in quiescently sedimenting samples (D—7 mg/mL ATP + 30 mM MgCl_2_, compared to 75 mg/mL ATP, both in the presence of 333 mg/mL PEG‐3350, pH 7).

### 2.4 The Effects of Adenosine Di‐ and Monophosphate and Different Crowder Types on Phase Separation and Droplet Formation

Additional experiments conducted in the course of this work have shown that both ADP and AMP (adenosine di‐ and monophosphate) also form droplets in the presence of concentrated PEG, although the droplet state is less stable at low pH in either case and tends to undergo liquid‐solid phase transition (LSPT), that is, crystallization (Figures S5 and S6). We also note that all three nucleotides can form droplets cooperatively when each of them is present separately in a subcritical concentration (Figure S7). Finally, it should be emphasized that although a change in the molecular weight of PEG has only a minor effect on LLPS in aqueous ATP solution, replacing PEG with the polysaccharide‐like crowding agents Ficoll‐70 or Dextran‐10 render the transition no longer observable under the typical set of physicochemical conditions (Figure S8). Hence, the pronounced surface hydrophobicity of PEG (and its hydrogen‐bond‐acceptor, rather than ‐donor, properties) in concert with the excluded volume effect must be responsible for triggering the phase separation.

## 3 Conclusion

In summary, we have discovered that ATP — the cell's central metabolite — has the intrinsic capacity to undergo liquid–liquid phase separation in the absence of proteins or peptides. The amphilic nature of ATP, presenting Coulombic, hydrophobic, π‐stacking and possibly also entropically favorable anion‐π interactions [60], permits stability and dynamics of the ATP droplets over a wide range of pH values and ionic strengths. Under physiologically relevant crowding conditions and with millimolar Mg^2^^+^, this phase separation occurs at intracellular ATP concentrations. These findings elevate ATP from a mere modulator of protein condensation to an autonomous phase‐separating species capable of forming its own condensates. Local increases or fluctuations in ATP and Mg^2^^+^ levels can therefore directly nucleate or regulate membraneless organelles, while dysregulated ATP phase behavior could contribute to pathological condensation pathways. This previously overlooked facet of ATP biochemistry opens new avenues for understanding cellular organization and the molecular origins of condensate‐related diseases.

## Conflicts of Interest

The authors declare no conflict of interest.

## Supporting Information

Experimental section; additional characterization and control experiments on the two‐phase ATP‐PEG system, as well as ADP‐PEG and AMP‐PEG systems.

## Supporting information




**Supporting file 1**: chem71374‐sup‐0001‐SuppMat.docx.

**Supporting file 2**: chem71374‐sup‐0002‐SImovie.zip.

## Data Availability

The data that support the findings of this study are available from the corresponding author upon reasonable request.
