# Supplementary material for: Protein‐Independent Liquid–Liquid Phase Separation of Adenosine Triphosphate Under Crowded Conditions
Source: Chemistry. 2026 Jul 3;32(38):e71374. doi: 10.1002/chem.71374 (PMC13660498; doi:10.1002/chem.71374)
Supplement: Supplementary file 1 [file CHEM-32-e71374-s002.docx]

**SUPPORTING INFORMATION**

for

**Protein-Independent Liquid–Liquid Phase Separation of Adenosine Triphosphate under Crowded Conditions**

Robert Dec^1, 2^, Wojciech Dzwolak^3*^, Roland Winter^1*^

^1^ Physical Chemistry I - Biophysical Chemistry, Department of Chemistry and Chemical Biology, TU Dortmund University, Otto-Hahn Strasse 4a, 44227 Dortmund, Germany

^2^ Present address: Biophysical Chemistry, Ruhr-Universität Bochum, Universitätsstraße 150, 44801 Bochum, Germany

^3^ Faculty of Chemistry, Biological and Chemical Research Centre, University of Warsaw, Pasteur Street 1, 02-093 Warsaw, Poland.

* Corresponding authors:

E-mails: wdzwolak@chem.uw.edu.pl, [roland.winter@tu-dortmund.de](mailto:roland.winter@tu-dortmund.de)

**CONTENTS**

1. Materials and Methods

Control microscopy images for OD measurements of droplet formation reported in Figure 1.

2. Stability of the aqueous two-phase ATP-PEG system at room temperature and reversibility of the LLPS process during heating/cooling cycles
3. Estimation of partition coefficients of quinacrine and PEG-FITC between the dense and light ATP-PEG phases.
4. Microscopy image of ATP-PEG droplets persistent at high salt concentration
5. LLPS and LSPT in the ADP-PEG system
6. LLPS and LSPT in the AMP-PEG system
7. LLPS in mixed ATP+ADP+AMP-PEG system
8. ATP droplets: PEG-3350 vs. other macromolecular crowders
9. Table S1. Estimation of the volume of the dense phase

**1. Material and Methods**

***Samples.*** PEG-2000 was purchased from Fluka Biochemika (Honeywell Research Chemicals), Dextran-10 from Carl Roth, Ficoll-70 from GE Healthcare Bio-Sciences AB, PEG-FITC (MW 3350) from Creative PEGWorks, the ATP determination kit from Invitrogen (Thermo Fisher Scientific). ATP (adenosine 5′-triphosphate disodium salt hydrate), ADP, AMP (disodium salt), PEG-3350, PEG-8000, quinacrine, NaCl, MgCl_2_ and all other reagents were purchased from MilliporeSigma (formerly Sigma-Aldrich). Samples were prepared by suspending and dissolving weighted amounts of ATP (or ADP/AMP) and crowder in water at approximately 90% of the targeted total volume, followed by pH-adjustment of stirred liquids with NaOH or HCl to the desired values followed by an addition of water to the total targeted volume and confirmation of the final pH value. The pH was therefore determined in the presence of ATP and PEG. Since the samples where pH was tested were prepared at very different pH levels, stabilising pH through the presence of a buffer would require the use of different buffers depending on the pH. The presence of different buffers would complicate the interpretation of the results, as different components of different buffers could interact with ATP in different ways.

In the case of NaCl or MgCl_2_-containing samples, the procedure was modified so that the specific salt was present in the sample at the desired concentration (the NaCl concentration given in the graphs does not take into account the concentration of ions introduced as a result of pH adjustment and ions present in disodium ATP salt).

***Optical microscopy.*** Light microscopy images were recorded on an Eclipse TE2000-U microscope (Nikon Inc.) equipped with a Nikon Plan Fluor 10x objective (0.45, WD 4.0) and additionally a X-Lite™-120 fluorescence Illumination System EXFO and a FITC-HQ filter (excitation 480/40, emission 527/30) for the PEG-FITC and quinacrine fluorescence mode. The maximum possible resolution was used for microscopy measurements (the photos shown in the figures are fragments of the original images). For measurements under normal light, brightness, gain, and exposure were set to automatic mode. For measurements under fluorescent light, gain and exposure were set to levels that ensured decent contrast without signal oversaturation.

***Optical density (turbidity) measurements.*** Optical density (OD) was measured at 500 nm using a UV-1800 Shimadzu UV Spectrophotometer in a 3 mm pathlength cuvette. Unless otherwise indicated, measurements were carried out at 21 °C. Samples were vortexed directly before the measurement (also in the case of measurements related to cooling and testing the effect of elevated temperatures). The OD measurements at elevated or decreased temperatures were performed on samples containing the highest tested concentrations of ATP and PEG (75 and 333 mg/mL, respectively), which at room temperature exhibit high optical density (Fig. 1D of the main manuscript). Because the ATP–PEG system revealed high sensitivity to elevated temperatures, we aimed to test whether cooling could induce LLPS in samples containing sub-threshold ATP (Fig. 1E, green trajectory) or PEG concentrations (Fig. 1E, black trajectory), in which abundant droplets are not present at room temperature. Wherever error bars are not explicitly presented in the main manuscript, the plots/trajectories shown represent typical experimental outcomes obtained in a highly reproducible manner.

***Circular dichroism (CD) spectroscopic measurements.*** For the CD measurements, the dense droplet phase on the ATP-PEG system (obtained at the specified pH) system was centrifuged and placed in a demountable quartz cuvette of 0.01 mm optical path. The CD spectrum, corrected for the buffer signal, was acquired at 25 ^o^C by accumulation of 20 independent spectra (at 200 nm/s scanning rate) on a J-815 S spectropolarimeter from Jasco Corp. (Tokyo, Japan).

**2. Control microscopy images for OD measurements of droplet formation reported in Figure 1.**

**Figure S1. Control microscopy images for OD measurements of droplet formation reported in Figure 1.** The images were collected for samples stored prior to the measurements at ambient conditions (A) or on ice (B). The sample conditions are indicated above the images.

**3. Stability of the aqueous two-phase ATP-PEG system at room temperature and reversibility of the LLPS process during heating/cooling cycles**

**Figure S2.** (A) Brightfield microscopy images of ATP-PEG-3350 droplets after 7 days at room temperature at different pH values (samples shaken before the measurement). Samples: 75 mg/mL ATP, 333 mg/mL PEG (average molecular weight 3350), H_2_O; pH as specified in the figure; room temperature (including during aging). (B) Reversibility of the LLPS process probed by OD measurements at 500 nm upon multiple heating (red arrows) and cooling (blue arrows) cycles between 20 ^o^C and 70 ^o^C. The microscopy image on the right corresponds to the sample after three consecutive heating/cooling cycles. Other conditions: 75 mg/mL ATP, 333 mg/mL PEG, pH 7.0, 3 mm optical pathway.

**4. Estimation of partition coefficients of quinacrine and PEG-FITC between the dense and light ATP-PEG phases**

**Figure S3.** **Estimation of partition coefficients of quinacrine and PEG-FITC between the dense and light ATP-PEG phases.** Top: calibration of quinacrine (left) and PEG-FITC (right) fluorescence intensities vs. fluorophore concentration; Bottom: calculated partition coefficients of both fluorophores.

Quinacrine was added to a sample containing 75 mg/mL ATP, 333 mg/mL PEG, and pH 7.0 at a concentration of 0.02 mg/mL. PEG-FITC was added at a concentration of 0.2 mg/mL. Thus, the samples were prepared in the same manner as the samples for the microscopy measurements, the results of which are shown in Fig. 2. Subsequently, both samples (with quinacrine and PEG-FITC) were left for 24 hours, during which time macro-phase separation occurred. Portions of the lower and upper phases were carefully collected and diluted to concentrations at which the measured fluorescence intensity fell within the range covered by the calibration curve. The measurement was performed using a plate reader. One well of the plate contained 70 µL of diluted sample (from the bottom or top phase). Three wells were allocated for each sample. Measurements were performed at room temperature. For quinacrine, an excitation wavelength of 425 nm was used, and emission was measured at 505 nm. For PEG-FITC, an excitation wavelength of 488 nm was used, and emission was recorded at 520 nm.

**5. Microscopy image of ATP-PEG droplets persistent at high salt concentration**

**Figure S4.** Brightfield microscopy image of the ATP-PEG system in the presence of extremely high salt concentration: 75 mg/mL ATP, 333 mg/mL PEG-3350, 3.5 M NaCl, pH 7.0; room temperature.

**6. LLPS and LSPT in the ADP-PEG system**

**Figure S5.** Brightfield microscopy images of the ADP-PEG-3350 system: droplets also form, but at very low pH, the system reveals a stronger tendency to form crystals. Samples: 75 mg/mL ADP, 333 mg/mL PEG (average molecular weight 3350), H₂O; pH as specified in the figure; room temperature.

**7. LLPS and LSPT in the AMP-PEG system**

**Figure S6.** Brightfield microscopy images of the AMP-PEG-3350 system: crystals or solid aggregates dominate at low pH, whereas droplets prevail at pH 7 and above. Samples: 75 mg/mL AMP, 333 mg/mL PEG (average molecular weight 3350), H₂O; pH as specified in the figure; room temperature.

**8. LLPS in the mixed ATP+ADP+AMP-PEG system**

**Figure S7.** ATP, ADP, and AMP in PEG at concentrations below their individual droplet-permissive concentration thresholds form droplets when mixed together. Comment: ATP, ADP, and AMP in PEG do not form light-scattering droplets when used at a concentration of 32.5 mg/mL (0.5× in the graphs). However, if samples contain simultaneously ATP and ADP (or ATP/AMP, ADP/AMP), each at 32.5 mg/mL, strong absorbance and numerous droplets are observed.

**9. ATP droplets: PEG-3350 *vs*. other macromolecular crowders**

**Figure S8.** Brightfield microscopy images of ATP in the presence of alternative macromolecular crowders. Droplets are still present in the ATP–PEG system when PEG-3350 is replaced by PEG-2000 or PEG-8000, but are absent if PEG is replaced with Dextran-10 or Ficoll-70. Comment: PEG, due to its amphiphilic character, can sequester water and enhance hydrophobic interactions. Dextran and Ficoll do not have this property. Samples: 75 mg/mL ATP, 333 mg/mL molecular crowder (Dextran-10, Ficoll-70, PEG-2000, or PEG-8000), H₂O; pH as specified in the figure; room temperature.

**10. Table S1. Estimation of the volume of the dense phase.**

**Table S1: Estimation of ATP dense phase volume (relative to the total volume) at different pH values based on the spectrophotometric data presented in Figure 2A.**

The volume fraction (f) of the bottom (dense) phase can be calculated based on the results of the spectrophotometric ATP concentration measurements of the bottom and upper phases and taking into the account that the average concentration for the entire sample volume is 75 mg/mL (75 = f⋅[ATP]^DENSE PHASE^ + (1-f)⋅ [ATP]^UPPER PHASE^ - based on the absorbance readouts shown in Fig. 2A). The higher ATP concentration in the condensed phase at lower pH likely reflects the fact that charge-depleting re-protonation of ATP at low pH decreases electrostatic repulsion between ATP molecules, allowing them and the surrounding water molecules to pack more densely, resulting in a more concentrated condensed phase (consistent with depletion-driven phase separation in the crowded PEG environment).
